# Pre-clinical evaluation of therapies to prevent or treat bone non-union: a systematic review protocol

**DOI:** 10.1186/s13643-015-0148-6

**Published:** 2015-11-12

**Authors:** Sarah K. Stewart, Philippa M. Bennett, Sarah A. Stapley, Janine Dretzke, Danai Bem, Jowan G. Penn-Barwell

**Affiliations:** Royal Centre for Defence Medicine, Queen Elizabeth Hospital, Birmingham, B15 2TH West Midlands UK

**Keywords:** Animal model, Pre-clinical trial, Non-union, Delayed union, Critical size defect, Fracture model, Novel therapy

## Abstract

**Background:**

Non-union of fractured bone is a major cause of morbidity in the orthopaedic population. Despite this, optimal management of non-union is still unclear and remains a significant clinical challenge. Research continues in animal models in an attempt to identify an effective clinical treatment. The proposed systematic review will evaluate current therapies of bone non-union in animal models, in order to identify those that may translate successfully to clinical therapies.

**Methods/design:**

The methodology for the systematic review will be in accordance with standard guidelines. All potential sources for pre-clinical studies will be interrogated and the search strategy written in conjunction with a specialist in this field. Data extraction will be conducted by two reviewers to minimise bias. Analysis will be predominantly qualitative because of the heterogeneity that is likely to exist between the studies. However, quantitative synthesis will be performed where homogeneity in a sub-group of studies exists. Quality assessment will be undertaken utilising a risk of bias tool.

**Discussion:**

To date, there has not been a systematic review addressing bone non-union therapies in animal models despite the plethora of pre-clinical research currently being undertaken. This protocol details and outlines the methodology and justification for such a review.

**Electronic supplementary material:**

The online version of this article (doi:10.1186/s13643-015-0148-6) contains supplementary material, which is available to authorized users.

## Background

Non-union is a condition where fractured bone does not heal. This can result in delayed recovery, re-hospitalisation, unplanned revision surgery and potentially, limb amputation [1, 2]. The rate of non-union can be as high as 30 % in the most severe injuries seen in the civilian setting, rising to 50 % when seen in injuries caused by military weapons [1–3].

A number of therapies including electromagnetic field stimulation, ultrasound, shockwave intervention, and bone morphogenetic protein therapy have been tested in the clinical setting, aimed at preventing or treating non-union. Moreover, they have been evaluated in meta-analysis of clinical trials [4–7]. However, these meta-analyses reveal that there is limited evidence of the efficacy of these therapies. Pre-clinical and translational work utilising animal models to develop more potent treatments therefore continues.

Animal models eliminate the confounders that beset clinical work, through standardisation of conditions within a model. However, there is little standardisation between models used by different researchers with a breadth of methodologies and outcome measures being employed to evaluate models of bone non-union [8–11]. Current pre-clinical work investigating new therapies for promoting bone healing is prolific, as identified in a scoping search of primary studies. Novel strategies that have yet to translate to clinical studies include integrating stem cells into a polymer scaffold embedded with hydroxyapatite particles to stimulate bioactivity [12], use of other growth factors such as stromal-cell derived factor and transforming growth factor to augment the effects of bone morphogenetic proteins [13], and novel synthetic bone substitutes [14].

To date, there has been no work to systematically examine and compare all the current pre-clinical and translational evidence to prevent and treat non-union, which is yet to be evaluated in clinical trials. Here, we present a protocol for a systematic review investigating the clinical challenge of bone non-union in animal models.

## Objective

To systematically collect and analyse published and unpublished evidence of therapies for preventing and treating non-union, which are yet to be translated to clinical trials.

## Methods

This protocol was written in accordance with the preferred reporting items for systematic reviews and meta-analysis protocols (PRISMA-P) guidelines [15]. Reporting of the full systematic review will take into account both the PRISMA guidelines [16] and guidelines specific for systematic reviews of animal studies [17]. The protocol has been registered and published on Collaborative Approach to Meta Analysis and Review of Animal Data from Experimental Studies (CAMARADES). The protocol was not registered with PROSPERO, as reviews on animal models were not eligible for inclusion at the time of writing.

### Searches

The following sources will be searched for primary studies:

- Bibliographic databases—PubMed and Embase via Ovid.
- The Science Citation Index for citation searching.
- The British Library’s Zetoc research database.
- Hand searching citation lists of included studies and relevant reviews.
- Hand searching of relevant conference proceedings i.e. Orthopaedic Research Society, Orthopaedic Trauma Association, Experimental Biology, Tissue Engineering and Regenerative Medicine Society.
- Contact with study authors and researchers of ongoing trials.

A combination of text and index terms relating to the condition (bone non-union) and population (animal model) will be used. Terms relating to intervention (e.g. stem cells, shock waves) will not be included. This is justified as emerging therapies for bone non-union are likely to be so novel that eligible papers may be missed in the search if intervention terms are included. Although this will undoubtedly produce a high yield of studies from the search, the sensitivity of the search (and therefore comprehensiveness of the systematic review) will be improved.

A filter for the retrieval of animal studies as described by de Vries et al. [18, 19] will be utilised. These have been shown to retrieve greater numbers of animal studies than the standard search filters on these engines. Language restrictions will not be applied. A date restriction (2004–present) will be applied. Justification for this decision stems from the likelihood that studies conducted prior to 2004 will either have already translated to clinical research (rendering them ineligible for inclusion in the systematic review), or found to confer insufficient benefit to warrant translation to the human population. An example search strategy is included in Additional file 1.

An electronic database (EndNote version X7.1, Thomson Reuters, New York) will be used to store and manage search results, regardless of whether the studies are eventually included or excluded in the systematic review. Titles and abstracts generated from the search will be screened by two reviewers (SKS/PMB) in order to establish if they potentially meet the inclusion criteria. Full articles will be obtained for all records that are identified as potentially meeting the inclusion criteria, or where there is uncertainty. Two reviewers (SKS/PMB) will independently screen and evaluate full text reports against the inclusion criteria. Differing of opinions between the two reviewers will be resolved by discussion with a third reviewer (JPB). Where ambiguity remains, contact with the study’s authors will be attempted to clarify issues pertaining to eligibility for the systematic review. Papers in languages other than English, whose abstracts indicate that they may meet the inclusion criteria, will be translated in full or in part. A PRISMA flow diagram will be used to document the study selection process, and a record of excluded studies (including reasons for exclusion) will be kept.

### Selection criteria

#### Types of studies

Only controlled trials will be eligible for inclusion. In vitro experiments, cohort studies, case reports, reviews and letters will be excluded. Given the nature and ease of randomisation in animal studies, it is likely that the majority of studies generated from the searches will be randomised controlled trials. Both unpublished and published works are eligible for inclusion.

#### Types of participants

Eligible studies must use a mammalian model to test an intervention to treat or prevent bony non-union. There will be no restrictions placed on the anatomical site of the bony defect created. Studies with induced co-morbidities (e.g. immunosuppression, osteoporosis) in the animal subject will be included.

#### Intervention

The study assesses an intervention in a mammalian animal model intended to

- Prevent non-union
- Treat non-union
- Promote or accelerate healing of a bony defect
- Treat or ameliorate delayed union

The intervention must be administered after formation of a bony defect in an animal model. There may be studies that test an established intervention but delivered to the animal model through a novel vehicle. These will be included as they may represent a more effective treatment modality.

#### Comparator

A group of control animals are described, which receive

- No treatment
- Current standard of care for the insult in question
- An alternative treatment to the intervention being analysed

#### Outcome measures

The primary outcome of interest is bone formation. A quantifiable measure of bone formation must therefore be assessed (morphometrical analysis). This can be achieved through radiological means and/or histological means.

Secondary outcomes of interest will be related to the structural properties of the newly formed bone (biomechanical analysis), which may include:

- Indentation testing
- Microtensile and microcompressive analysis
- Three- and four-point bending

Papers that perform biomechanical analysis (secondary outcomes) in the absence of morphometrical analysis (primary outcomes) will not be included in the systematic review.

#### Exclusion of therapies with evidence of clinical evaluation

The stated intention of this study is to examine emerging therapies that have not yet been evaluated clinically. To that end, studies describing therapies that have translated into clinical studies will be filtered. The authors will exclude all papers detailing interventions that they know are already in clinical trials or indeed being used in clinical practice. Where uncertainty exists, the following steps will be performed prior to data extraction:

- PubMed and Embase will be searched for clinical studies that cite each study that otherwise meets the inclusion criteria.
- PubMed and Embase will be searched using keywords describing the specific therapies for studies describing clinical evaluation.

All studies describing therapies that are found to have been evaluated clinically will be excluded, and the results of this ‘clinical filter’ will added to the PRISMA flow chart.

### Data extraction

A proforma detailing the necessary information to be extracted from the eligible articles has been designed (Additional file 2). The proforma will be piloted by the two reviewers (SKS/PMB) prior to commencing the data collection formally. This will serve to not only test the efficacy of the proforma itself, but also calibrate the reviewers to ensure consistency. Information required (but not limited to) from each study is detailed in Table 1.

**Table 1:** Suggested data to be extracted from eligible papers

Publication details	Authors, year of study, year of publication, geographical location of study, language of paper, funding sources
Study design and quality	Sample size, *p* values, methods to randomise, allocation concealment, blinding of outcome assessment.
Model of non-union	Non-union vs delayed union
Species of animal	Mouse, rat, etc.
Characteristics of animal	Age, weight, strain, sex, genetic modification, co-morbidities
Nature of insult	Location of bony insult (tibia, femur, etc.), fracture model (segmental vs non-segmental), osteotomy technique, size of osteotomy, closed vs open, critical vs non-critical size defect, fixation vs no fixation, internal vs external fixation (if fixation used), isolated or multiple locations,
Additional insults (other than bony defects)	Infection, contamination, immunosuppression, soft tissue injury, vascular injury.
Nature of intervention	Type of intervention (pharmacological vs non-pharmacological), time point that intervention was applied to animal model post-fracture creation, frequency of intervention (single vs recurrent), duration of intervention, systemic or local dosing
Duration of follow-up	Single vs multiple time points of follow-up
Outcome measures	Morphometrical analysis (e.g. histology, radiology), biomechanical analysis (e.g. torsional stiffness)
Results	Main findings, direction of effect, effect sizes and uncertainty, statistical significance, length and adequacy of follow-up, early animal death
Additional insults (other than bony defects)	Infection, contamination, immunosuppression, soft tissue injury, vascular injury.

### Missing data

During proforma completion, we will seek missing data that is required for analysis from the original study author. If a study does not mention an item detailed on the data collection form and the review authors are unable to obtain the information, its absence will be noted and presumed not to have been performed (e.g. no mention of randomisation will be presumed that it was not performed). This domain will be labelled as ‘high risk’ and its consequential impact on the validity of the study will be assessed when conducting risk of bias assessment (see below).

### Quality assessment

Potential bias within the pre-clinical studies will be determined by the reviewers prior to analysis. Both the Cochrane Collaboration tool [20] and Systematic Review Centre for Laboratory animal Experimentation’s (SYRCLE) risk of bias tool [21] which is specific for animal studies will be used. Criteria such as sequence generation, allocation concealment, blinded outcome assessment and incomplete outcome data (e.g. animals that died prematurely) will be identified and recorded, and each domain given a rating of ‘high risk’ or ‘low risk’ based on the details provided in the literature. Where information is missing, the domain will be labelled as ‘unclear’.

## Analysis

Results from this systematic review will be presented in the form of a narrative synthesis. Key study characteristics such as animal species, intervention and results will be tabulated to aid clarity of study findings.

In the instance where homogeneity exists between a number of studies, quantitative synthesis will be attempted using Review Manager (RevMan v5.3) software. Whilst the generation of pooled effect sizes may not be as meaningful compared to ones provided by clinical trials, meta-analyses of animal studies may still be useful for showing the overall direction of effect and the heterogeneity across studies. Moreover, they may serve to generate hypotheses [22].

### Sub-group analysis

Sub-group analysis will be considered where studies can be grouped according to the following characteristics:

- Animal species (e.g. mice, rats, other mammalian species)
- Animal sex (e.g. male, female, mixed)
- Experimental intervention used (e.g. autografts, allografts, synthetic)
- Delivery of experimental intervention (e.g. oral, intravenously, directly to fracture site).
- Nature of defect created (critical or non-critical defect)
- Models of non-union (hypertrophic or atrophic)
- Models of delayed union versus non-union
- Prevention versus treatment of non-union
- Application of an insult (e.g. osteoporosis, infection, diabetes)
- Methodological quality according to Cochrane risk of bias tool (e.g. by individual domain)

It is recognised that a large number of sub-group analyses on small numbers of studies may affect the power of the analyses; should this be the case here, any findings from sub-group analyses will have to be interpreted cautiously. Presentation of (separate sets of) results in forest plots without the calculation of a summary measure may also be considered in order to highlight any heterogeneity across studies.

### Effect measures for continuous outcomes

The primary outcome measure is bone formation. This will predominantly be in the form of continuous data, for example, bone mass densities (mg m^−2^). In these instances, the mean difference between formation of bone in the experimental and control groups will likely be presented, and forest plots can be used to show the pooled mean difference. The standardised mean difference may also be used in instances where papers are measuring the same outcome, e.g. bone density, but using different modalities to achieve this, e.g. micro-CT, photon emission CT, and X-ray.

### Effect measures for dichotomous outcomes

In studies which present dichotomous data, the relative risk may be presented and meta-analysis may enable the calculation of a pooled relative risk. A situation where this could occur is analysis of whether bone formation did or did not occur at a set time point. In studies which measure the time to the formation of new bone in the in vivo model, i.e. time-to-event data, the pooled hazard ratio may be calculable.

Given that residual heterogeneity is anticipated even between studies considered to be similar enough to pool, a random effects model (as opposed to a fixed effects model) is more likely to be appropriate for meta-analysis. A random effects model will therefore be used for quantitative analysis of dichotomous data. A random effects model has been chosen because it is based on the assumption that although the studies are different, they are estimating different, yet related intervention effects. If heterogeneity does exist, it will lead to a wider confidence intervals and therefore corresponding claims of statistical significance will be more conservative [23]

A decision on whether to pool will be made on the basis of an assessment of clinical and methodological heterogeneity. However, statistical heterogeneity will also be reported using the I^2^ statistic and the χ^2^ test. The likelihood of publication bias will be investigated through the construction of funnel plots for each meta-analysis containing ten or more studies.

### Assessment of bias

Assessing and analysing risk of bias will be an important component of the systematic review. Criteria determining the likelihood of bias will be collected as described above (see ‘Quality Assessment’) and a ‘risk of bias graph’ used to illustrate where bias predominates across studies. This will serve to inform the authors of where caution must be heeded when analysing the data. It is anticipated that a narrative discussion on the risk of bias will be incorporated into the review, included in which will be discussion on domains which consistently displayed ‘low’, ‘high’ or ‘unclear’ risk. Analysis as to why this may be the case and how this could affect translation of the pre-clinical trial to clinical trials will be discussed.

Where quantitative analysis of subgroups is possible, the threshold based on key bias domains that have been identified during the initial trawl will determine which studies are eligible for inclusion into the subgroup analysis. Key bias domains are derived from the CAMARADES quality checklist for animal studies [24], and include seven domains:

- Publication in peer reviewed journal
- Randomisation of treatment or control
- Allocation concealment
- Blinded assessment of outcome
- Sample-size calculation
- Statement of compliance with regulatory requirements
- Statement regarding possible conflict of interest

It is anticipated that studies at low risk of bias for the key domains will not be combined with studies at high or unclear bias for meta-analysis. In the event where such a priori criteria generate insufficient studies to perform quantitative analysis, pooling of studies regardless of bias risk may have to occur. In this instance, authors will highlight the reasoning for this in the review to ensure transparency of results.

### Control group bias

Due care in data synthesis must also be paid when studies share control groups between experimental groups. For example, a study may have a single control group but three different experimental groups with varying sizes of segmental defects created. When such comparisons are made, animals in the control group are essentially counted three times and the comparisons will receive too much weight in the estimation of summary effect [22]. Techniques such as splitting the control group to equalize the number of experimental groups before performing analysis [23], will be used to mitigate against this potential source of bias.

## Discussion

Systematic reviews of pre-clinical studies enable appraisal and analysis of potential treatments prior to translation to human studies. Although this protocol details the methodology for a systematic review of pre-clinical studies investigating bone non-union, other clinical challenges can use such an approach to the same effect.

In the absence of a widely established, effective treatment for bone non-union, and a lack of systematic reviews of all potentially relevant emerging treatments from the pre-clinical field, a new systematic review is clearly warranted. It is likely that this will be the most comprehensive to date in the area of novel and emerging treatments for bone healing.

A recent review by Mills and Simpson [11] examined the heterogeneity that exists between animal models for non-union, identifying a number of clinical scenarios which animal studies aim to reproduce. Even within subgroups of outwardly similar study designs, the variability between studies was extensive. As such, careful consideration and differentiation of the experimental question of studies included in the review is required. Based on their review, the following recommendations are suggested when sub-grouping papers for analysis.

Of particular note, delayed union is a distinct entity and must be differentiated from non-union in the systematic review to ensure transparency of analysis. Delayed union is prolonged bony healing compared to that of a control group, and will often be the pre-cursor to a non-union model. Unlike a model of non-union where the occurrence of such a defect can be objectively assessed at a set time point, delayed union is a clinical diagnosis with no defined criteria for assessment. Due to the fundamental differences in the definition of delayed union and non-union, separate syntheses will be performed.

Models where a segmental defect has been established should be considered separately to those where there is no iatrogenic bone removal. Attention during data synthesis is also required for models which involve high-energy trauma, open or comminuted fractures, and introduction of infection or immunocompromise, in addition to an intervention intended to treat or prevent non-union. For the purposes of meta-analyses, studies where there has been introduction of such an insult will be analysed separately and not combined with studies where an insult has not been introduced. Inclusion of an insult in an animal model of non-union will inherently produce higher rates of delayed and non-union, compared with those models where there is an intervention alone.

Finally, it is important to consider the difference between atrophic and hypertrophic models of non-union. Atrophic models are characterised by a failure of periosteal and endosteal activity, with minimal callus formation. Hypertrophic non-union is less common, and is typified by high callus formation and endochondral growth at the fracture sites. Given their contrasting pathophysiological processes, studies with hypertrophic and atrophic models will not be synthesised together.

As demonstrated in Mills and Simpson’s review, the heterogeneic nature of animal models will likely hamper interpretation of results across all studies. Variations in species, methodology and study characteristics may also limit the number of trials considered similar enough to meta-analyse, and sub-group analyses within these sets of studies may also not be possible. However, it is anticipated that by presenting all the available pre-clinical evidence, it will be possible to observe trends for effectiveness for one or more emerging techniques. Pooled effect sizes may not be meaningful in terms of their applicability to human populations, and any findings must be interpreted in the context of study quality and the extent to which they are considered translatable to other settings. However, this systematic review will be valuable for hypothesis generating and may be able to aid in the identification of suitable candidate treatments to take forward into clinical trials. Accurate and valid hypotheses can consequently be drawn as to which emerging therapies for bony non-union may translate successfully to the human population.

## Additional files

Additional file 1:
**Sample search strategy for PubMed.** (DOCX 15 kb) Additional file 2:
**Data collection form.** (DOCX 30 kb)

## Abbreviations

CAMARADES: Collaborative Approach to Meta Analysis and Review of Animal Data from Experimental Studies
PRISMA: Preferred Reporting Items for Systematic Reviews and Meta-Analysis
PRISMA-P: Preferred Reporting Items for Systematic Reviews and Meta-Analysis Protocols
SYRCLE: Systematic Review Centre for Laboratory animal Experimentation
