# Supplementary material for: Pre-clinical evaluation of therapies to prevent or treat bone non-union: a systematic review protocol
Source: Syst Rev. 2015 Nov 12;4:161. doi: 10.1186/s13643-015-0148-6 (PMC4643533; doi:10.1186/s13643-015-0148-6)
Supplement: Additional file 1: — Sample search strategy for PubMed. (DOCX 15 kb) [file 13643_2015_148_MOESM1_ESM.docx]

**Additional File 1: Sample search strategy for PUBMED**

1. exp Animals/
2. (animal$).mp.
3. Exp Mammals/
4. (mammal$).mp.
5. (murine or mouse or mice).mp.
6. (sheep or ovine).mp.
7. (rat$ or rodent).mp.
8. (dog$ or canine).mp.
9. (cat$ or feline).mp.
10. (pig$ or porcine or swine).mp.
11. (rabbit$).mp.
12. (goat$ or caprine).mp.
13. (monkey$ or chimp$ or ape).mp.
14. (pre-clinical OR preclinical).mp.
15. exp models, animal/
16. exp animal experimentation/
17. 1 or 2 or 3 or 4 or 5 or 6 or 7 or 8 or 9 or 10 or 11 or 12 or 13 or 14 or 15 or 16
18. exp fracture, bone/
19. exp fracture, healing/
20. exp bone regeneration/
21. (Nonunion or non-union).mp.
22. (Bone union).mp.
23. (Bony defect$ or bone$ defect).mp.
24. (Bony fracture or bone$ fracture).mp.
25. (Bony repair or bone$ repair).mp.
26. (Bony injury or bone$ injury).mp.
27. (Critical defect or critical size defect or critical-size defect).mp.
28. (non-healing defect or non healing defect).mp.
29. (Segment$ defect or non-segmental defect or nonsegmental defect).mp.
30. (Bone regeneration or bony regeneration).mp.
31. (Bone healing or bony healing).mp.
32. (Delay$ union).mp.
33. 18 or 19 or 20 or 21 or 22 or 23 or 24 or 25 or 26 or 27 or 28 or 29 or 30 or 31 or 32
34. 17 or 33
35. limit 33 to year="2001 -Current"

Exp denotes explode

‘$’ denotes truncation

.mp. denotes a search of title, original title, abstract, name of substance word and subject heading word.
