# Supplementary material for: Pre-clinical evaluation of therapies to prevent or treat bone non-union: a systematic review protocol
Source: Syst Rev. 2015 Nov 12;4:161. doi: 10.1186/s13643-015-0148-6 (PMC4643533; doi:10.1186/s13643-015-0148-6)
Supplement: Additional file 2: — Data collection form. (DOCX 30 kb) [file 13643_2015_148_MOESM2_ESM.docx]

**Additional File 2: Data Collection Form**

**DEMOGRAPHICS**

Form Version and date (*e.g. v1 01 Apr 15):*

Name of review author:

Date form completed:

Notes *(unpublished – for own use, e.g. references to be followed up):*

**METHODS**

**Details of Study**

Aim of intervention *(As stated in the trial report. What was the problem that this intervention was designed to address?):*

Aim of study *(As stated in the trial report. What was the trial designed to assess?):*

Model of non-union:

- Hypertrophic □ Atrophic □
- Non-union □ Delayed union □ Other *(e.g. acceleration of healing):* □

*If delayed/non-union, specify prevention or treatment:*

- Prevention □ Treatment □ Both □

Ethical approval:

Yes □ No □ Unclear □

Funding *(including source, amount if stated):*

**Participants**

Species *(including strain if applicable)*

Age

Weight

Sex

Male □ Female □ Mixed □ Not detailed □

Geographical location *(centre and country):*

**Animal characteristics**

Species, age, weight, strain, sex:

Genetic Modification:

Yes □ No □

*If yes, provide details:*

Induced Co-morbidities

Yes □ No □

*If yes, provide details:*

**Details of bony insult**

Anatomical location:

Single or multiple defect:

Single □ Multiple □

*If multiple, specify other locations:*

Fracture model:

- Segmental □ Non-segmental □
- Closed □ Open □
- Critical □ Non-critical □ Not specified □

Technique to produce bony defect *e.g. osteotomy:*

Size of osteotomy *(if segmental defect):*

Fixation used?:

Yes □ No □

Internal or external fixation *(if applicable):*

Internal □ External □

*Details of fixation* *e.g. product/brand name, type of plate and screws:*

Additional Insults (other than bony defect) *e.g. infection, associated soft tissue injury:*

Yes □ No □

*If yes, provide details:*

**Nature of Intervention**

Details of Intervention *(including theoretical basis and references):*

Details of Co-interventions *(i.e. interventions separate to the intervention of interest):*

Details of control *(e.g. no treatment given, standard treatment given):*

**Delivery of Intervention** *(for each intervention in the study e.g. Intervention A, Intervention B)*

Local or systemic (to bony insult):

Local □ Systemic □

Timing of application of intervention *(e.g. immediately, 24 hrs post bony defect):*

Frequency of intervention:

Single □ Recurrent □

*If recurrent, specify frequency/number of applications:*

Duration of Intervention:

**Intervention Quality**

Fidelity/Integrity *(i.e. was the intervention delivered as intended? Evidence base for the intervention?):*

**OUTCOMES**

**Primary Outcome Measure**

Histological □ Radiological □ Both □

Modality of primary outcome measure *(e.g. CT scan)*- if answered ‘both’ above, provide details of each:

Timing of primary outcome measure (*including frequency and length of follow up) -* if answered ‘both’ above, provide details of each:

**Secondary Outcome Measure**

Yes □ No □

Modality of secondary outcome measure, if applicable (*e.g. three point bending)*:

Timing of secondary outcome measure, if applicable:

**Adverse events *(e.g. unexpected death of animal):***

**RESULTS**

Complete on attached Excel spreadsheet

**ASSESSMENT OF RISK OF BIAS**

Adapted from ‘SYRCLE risk of bias tool for animal studies’ [28] by Hooijmans *et al.*

| **DOMAIN** | **REVIEW AUTHOR’S JUDGEMENT** | **SUPPORT FOR JUDGEMENT** |
| --- | --- | --- |
| Sequence generation | High Risk □  Unclear □  Low Risk □ | Describe the methods used, if any, to generate the allocation sequence in sufficient detail to allow an assessment whether it should produce comparable groups. |
| Baseline characteristics | High Risk □  Unclear □  Low Risk □ | Describe all the possible prognostic factors or animal characteristics, if any, that are compared in order to judge whether or not intervention and control groups were similar at the start of the experiment. |
| Allocation concealment | High Risk □  Unclear □  Low Risk □ | Describe the method used to conceal the allocation sequence in sufficient detail to determine whether intervention allocations could have been foreseen before and during enrolment. |
| Random housing | High Risk □  Unclear □  Low Risk □ | Describe all measures used, if any, to house the animals randomly within the animal room. |
| Blinding (intervention) | High Risk □  Unclear □  Low Risk □ | Describe all measures used, if any, to blind trial staff from knowing which intervention each animal received. Provide any information relating to whether the intended blinding was effective. |
| Random outcome assessment | High Risk □  Unclear □  Low Risk □ | Describe whether or not animals were selected at random for outcome assessment, and which methods to select the animals, if any, were used |
| Blinding (outcome) | High Risk □  Unclear □  Low Risk □ | Describe all measures used, if any, to blind the outcome assessors from knowing which intervention each animal received. Provide any information relating to whether the intended blinding was effective. |
| Incomplete data outcome | High Risk □  Unclear □  Low Risk □ | Describe the completeness of outcome data for each main outcome, including attrition and exclusions from the analysis. State whether attrition and exclusions were reported, the numbers in each intervention group (compared with total randomized animals), reasons for attrition or exclusions, and any re-inclusions in analyses for the review. |
| Selective outcome reporting | High Risk □  Unclear □  Low Risk □ | State how selective outcome reporting was examined and what was found. |
| Other sources of bias | High Risk □  Unclear □  Low Risk □ | State any important concerns about bias not covered by other domains in the tool. |

**NOTES**

Contact with author attempted:
 Yes □ No □

If yes, information obtained?:

Yes □ No □

Was the study translated from a language other than English?

Yes □ No □

If yes, is further translation required *(i.e. of full text)*

Yes □ No □
